# Universality of Thermodynamic Constants Governing Biological Growth Rates

**DOI:** 10.1371/journal.pone.0032003

**Published:** 2012-02-14

**Authors:** Ross Corkrey, June Olley, David Ratkowsky, Tom McMeekin, Tom Ross

**Affiliations:** Tasmanian Institute of Agriculture/School of Agricultural Science, University of Tasmania, Hobart, Tasmania, Australia; University of South Florida College of Medicine, United States of America

## Abstract

**Background:**

Mathematical models exist that quantify the effect of temperature on poikilotherm growth rate. One family of such models assumes a single rate-limiting ‘master reaction’ using terms describing the temperature-dependent denaturation of the reaction's enzyme. We consider whether such a model can describe growth in each domain of life.

**Methodology/Principal Findings:**

A new model based on this assumption and using a hierarchical Bayesian approach fits simultaneously 95 data sets for temperature-related growth rates of diverse microorganisms from all three domains of life, Bacteria, Archaea and Eukarya. Remarkably, the model produces credible estimates of fundamental thermodynamic parameters describing protein thermal stability predicted over 20 years ago.

**Conclusions/Significance:**

The analysis lends support to the concept of universal thermodynamic limits to microbial growth rate dictated by protein thermal stability that in turn govern biological rates. This suggests that the thermal stability of proteins is a unifying property in the evolution and adaptation of life on earth. The fundamental nature of this conclusion has importance for many fields of study including microbiology, protein chemistry, thermal biology, and ecological theory including, for example, the influence of the vast microbial biomass and activity in the biosphere that is poorly described in current climate models.

## Introduction

Temperature governs the rate of chemical reactions including those controlling the development and decline of life on earth from individual cells to complex populations. This has been so across geological time and will continue to be so as the world faces unprecedented uncertainty reflected in popular, political and scientific debate about the impact of temperature on the biosphere. Models to describe the effect of temperature on biological systems reliably are becoming increasingly important. One such family of models [1]–[2] assumes a single rate-limiting, enzyme-catalyzed ‘master reaction’ using an Arrhenius form modified by terms that describe the temperature-dependent denaturation of that enzyme. Earlier [3] we presented such a model that specifically describes the Gibbs free-energy change upon folding/unfolding of the putative master enzyme as a function of temperature and individually fitted it to 35 bacterial strains, but the approach required three parameters to be held fixed (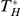, 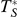, and 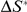). Here we begin with the same assumption but use a Bayesian hierarchical modeling approach to analyze simultaneously 95 data sets from all three domains of life, Bacteria, Archaea and Eukarya [4], and, with only minimal constraints, estimate all thermodynamic parameters, some of which were previously only accessible by experiment. Importantly, the model implies that the temperature-related growth rate of all poikilotherm life behaves as if controlled by a single-enzyme system and the growth range is constrained by the denaturation of that enzyme.

## Methods

### Data

The data comprise standardized growth rates (or rates of metabolism in some cases) of 95 strains from 19 Bacteria, 19 Archaea, and 13 unicellular Eukarya species (Table S1). Here we use the yeasts to represent the domain Eukarya. The data were obtained by an *ad hoc* process, but are sufficiently diverse in origin and species to enable us to test the thermodynamic model's validity for physiologically different organisms and included observations at temperatures below and above the maximum growth rate. They include psychrophiles (*e.g. Gelidibacter* sp.), psychrotrophs (*e.g. Shewanella gelidimarina*), mesophiles (*e.g. Escherichia coli*), thermophiles (*e.g. Streptococcus thermophilus*), acidophiles (*e.g. Ferroplasma acidiphilum*), halophiles (*e.g. Haloarcula vallismortis*) and haloalkaliphiles (*e.g. Natronococcus occultus*).

### Model Structure

Our model (equations 1–2) assumes that the growth rate is governed by a single enzymic rate-controlling reaction system that is limiting under all conditions. The quantity 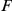 is the growth rate given the temperature and the values of the parameters. The numerator is essentially an Arrhenius model that describes the rate of the putative enzyme-catalyzed rate-controlling reaction (RCR) as a function of temperature while the denominator (equation 2) models the change in expected rate due to the effects of temperature on the conformation and activity of the putative enzyme catalyzing that reaction and is essentially the reciprocal of the probability, as a function of temperature, that the enzyme is in a catalytically active conformation.
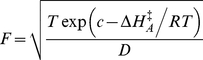
(1)where:
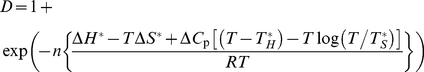
(2)


In the model R is the gas constant (8.314 J/K mol); 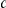 is a scaling constant; 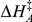 the enthalpy of activation (J/mol) of the rate limiting reaction; 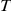 is the temperature (K); 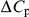 is the heat capacity change (J/K mol-amino acid-residue) upon denaturation of the RCR; 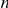 is the number of amino acid residues in the RCR; 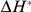 (J/mol-amino acid-residue) is the enthalpy change at 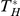, the convergence temperature for enthalpy (K) of protein unfolding; and 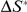 (J/K mol-amino-acid-residue) is the entropy change at 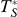, the convergence temperature for entropy (K) for protein unfolding. We derive two further quantities. One is the average number of non-polar hydrogen atoms per amino acid residue [5]: 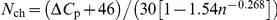. The 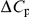 is associated with the energy required to reorganise the water molecules surrounding the protein [6] and increases with the non-polar accessible area of the molecule [7], as measured by 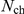. The other is the temperature at which 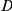 in equation 2 reaches a minimum and at which denaturation is minimized [3]: 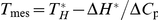
which provides an index of temperature adaptation of the organism. While the temperature of maximum growth could be used as an index, 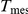 is a more natural choice since it is the temperature at which the enzymic rate-controlling reaction system has evolved to be maximally active. At higher temperatures the growth rate may increase, but enzymic activity declines.

We allow four parameters to have values specific to each strain 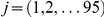: 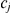, 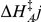, 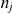, and 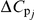. We assume the strain parameters to be Gaussian distributed with means specific to their domains: Bacteria, Archaea, or Eukarya. The remaining parameters (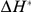
_,_ 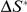, 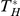, and 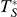) have values common to all strains and are thought not to depend on the individual biochemistry of each strain, but describe protein thermal stability limits [8]–[10]. In earlier analyses (data not shown) when data sets were grouped according to domain, estimates of the four thermodynamic constants related to protein stability were not significantly different between data sets and subsequently these were assumed to be universal, having the same values across all domains. This assumption is not disproven by the results and is supported by their narrow credible intervals. The common and domain parameters are each assigned a uniform prior with limits informed by the biochemistry literature. We assume that the square root of the observed growth rate has a Gaussian distribution with a mean given by the modeled value, 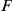, and with an unknown precision (reciprocal variance).

### Implementation

We use a Bayesian approach to allow for uncertainty in measurement and parameters to be incorporated in a natural way through the appropriate prior specification. We assign truncated normal priors to the strain parameters in which the means are specific to the domain of each, 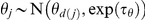, in which 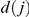 is the domain (Bacteria, Archaea, Eukarya) for strain 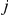. The 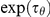 is the strain precision and models the variation between the strain parameters about the domain parameters, 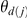. The domain means and the 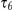 are assigned uniform priors with limits informed by the biochemistry literature with the exception of 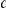 which is assigned a vague prior. The common parameters are each assigned a uniform prior with limits informed by the biochemistry literature. Finally, the observational precision is assigned a gamma distribution, 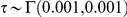. Prior specifications are documented in Table S2. Inference is obtained in the form of posterior means and variances using Markov Chain Monte Carlo (MCMC) simulation [11]. We choose to update the parameters of each strain as a block using Haario updates [12]. We also use Haario updates [12] for each set of domain parameters and the strain parameter precisions. We use separate Metropolis-Hastings [13] updates for each of the common parameters but to ensure rapid mixing we also use a Metropolis-coupled MCMC approach [14] based on 60 chains. During each iteration we randomly select a pair of chains 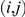 and swap their common parameters as a block with probability 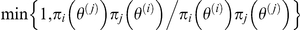, keeping other parameters unchanged. The model is run for 600,000 iterations and the last 50% of iterations are retained for further analysis. We summarize parameters using posterior block means, standard deviations, and 95% highest posterior density intervals (HPDI). A 95% HPDI is the shortest interval that contains a parameter with 95% probability. We determine the most likely ordering of the parameters in terms of their posterior probabilities by calculating the mean number of MCMC iterations for which each alternative arises.

## Results

**Figure 1:**
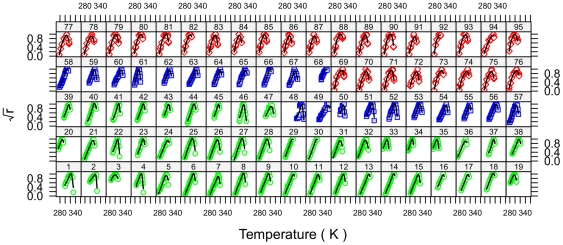
Observed and predicted growth rates by strain. Observed square root growth rate data are shown as circles and are standardized by dividing by the maximum for each strain. Fitted curves are shown as lines. Strain numbering is given in Table S1. Bacterial strains are shown as green circles, archaeal strains as blue squares, and eukaryote strains as red diamonds.

**Figure 2:**
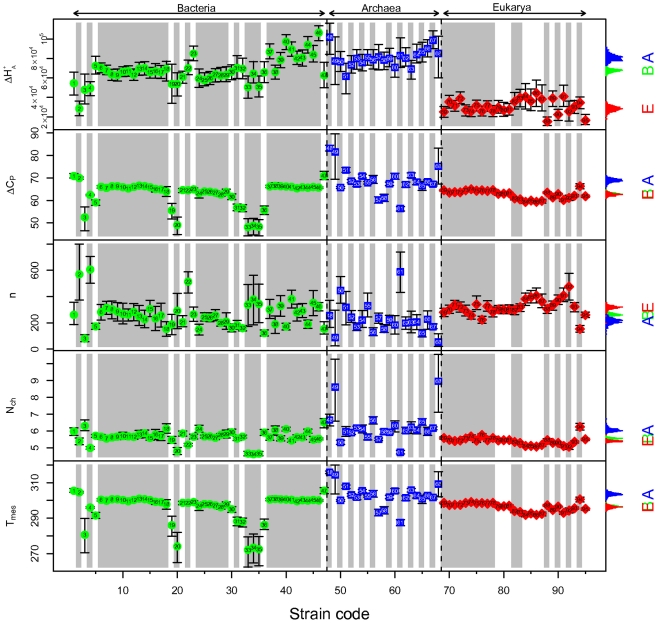
Posterior statistics for selected parameters. Posterior means and 95% HPDI are shown for 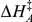, 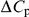, 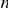, 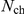, and 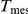. Bacterial strains are shown as circles, archaeal strains as squares, and eukaryote strains as diamonds. Posterior domain distributions are shown on the right margin. For both symbols and marginal distributions domains are colored green for Bacteria, blue for Archaea, and red for Eukarya. The strains are arranged so that those belonging to the same species are grouped contiguously. We show this by vertical gray and white shading that indicate when the strain species change; for example, strains 6—18 are all *E. coli*.

**Figure 3:**
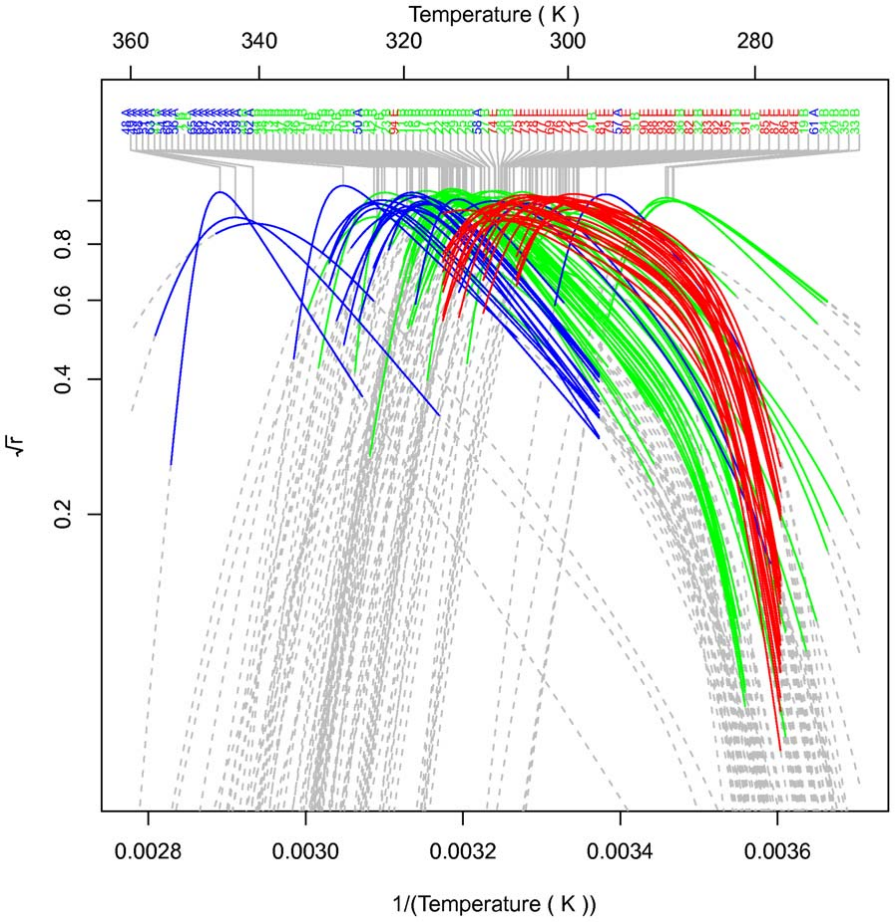
Fitted curves for growth rate by strain. Shown are the mean predicted growth curves plotted on a vertical log scale against the reciprocal of temperature. The colored portions indicate the observed temperature ranges for each strain, and the dashed portions are extrapolations outside these ranges. The curves are labeled with strain codes and domain. The domains are colored green for Bacteria, blue for Archaea, red for Eukarya.

**Figure 4:**
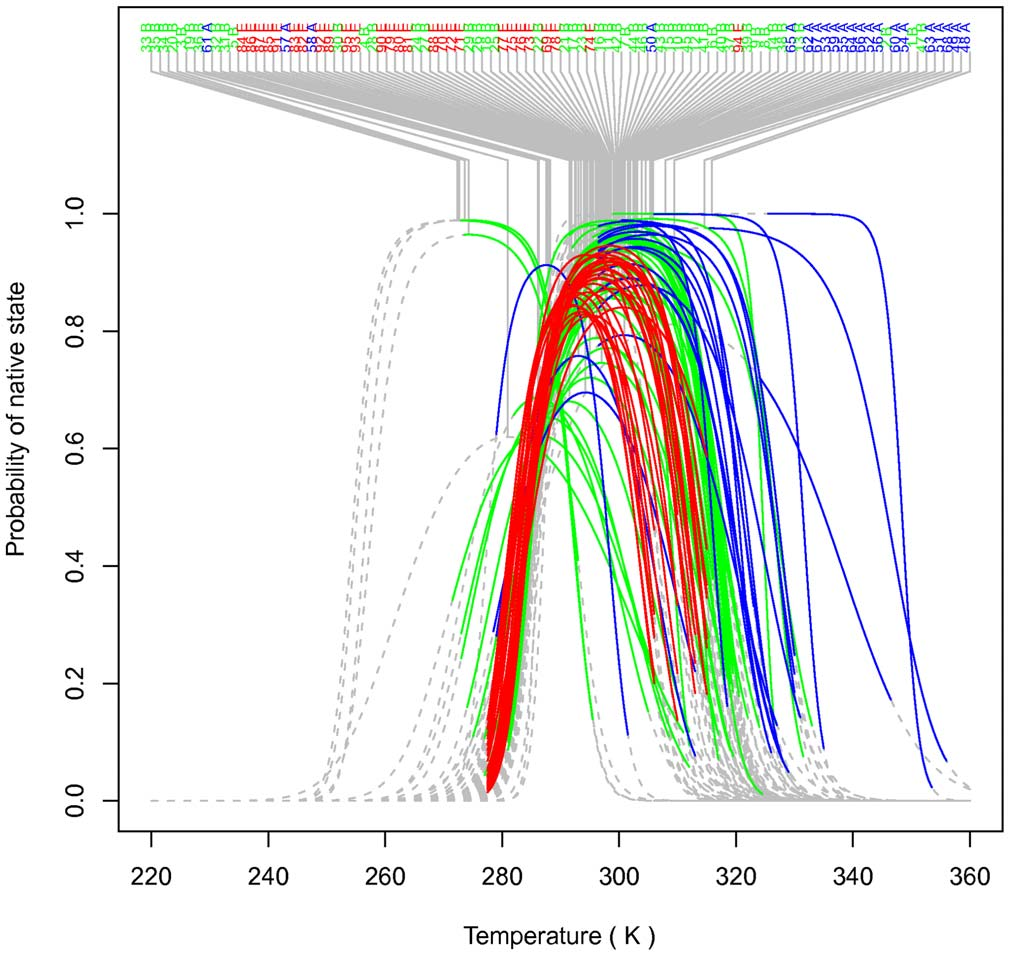
The probability of an enzyme being in its native state. Shown is the mean curve for each strain. The colored portions indicate the observed temperature ranges for each strain, and the dashed portions are extrapolations outside these ranges in order to show their shapes. The curves are labeled with strain codes and domain. The domains are colored green for Bacteria, blue for Archaea, red for Eukarya.

We obtain good fits for the growth curves of all strains as shown in Figure 1, and in more detail in Figures S1, S2, S3, S4, S5, S6. We summarize the strain parameters in Table S1 and Figure 2, common parameters in Table 1, and domain parameters in Table 2. Some strains appear outlying since they are below the 2.5^th^ or above the 97.5^th^ quantiles; *e.g.* ignoring domain, strains 46, 48, 67, 88, 90, 95 for 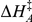, strains 33–35, 48, 49, 68 for 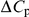, strains 2–4, 49, 61, 68 for 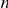, and strains 33–35, 48, 49, 68 for 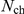. We show the fitted curves for all strains together in Figure 3 in which most of the strains with outlying parameters also have maxima that are either much lower than other strains (*e.g.* strains 20, 33, 35), or much higher (*e.g.* strains 48, 49, 68). This is also shown by the values of 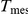 for the strains 33, 35, 20, which are lower than other strains (Figure 2). Some outliers may be explained when it is realized that those strains are at the extremes of thermal adaptation, such as strain 46, a thermophile, strain 48, a thermoacidophile, or strain 61, a psychrophile. In Figure 2, the strains 3, 19, 20, 33–35, appear to be outliers for 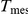; this is likely due to a lack of data in the lower temperature region, which also resulted in their wider credible intervals. Other variations in the data presented in Figure 2 are explicable after careful examination of Figures 1, 3, 4, and S1, 2, S3, S4, S5, S6.

**Table 1:** Posterior mean common parameter estimates.

Parameter	Mean (95% HPDI)
Enthalpy change (J/mol-amino acid-residue), 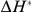	4970(4705, 5218)
Entropy change (J/K mol-amino-acid-residue), 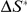	17.2 (16.3, 18.0)
Convergence temperature for enthalpy (K), 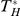	375.6 (371.4, 379.5)
Convergence temperature for entropy (K), 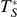	390.2 (384.7, 395.2)

**Table 2:** Posterior mean parameter domain estimates with 95% HPDI.

	Bacteria	Archaea	Eukarya
Enthalpy of activation (J/mol), 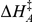	67549 (64887, 70312)	80799 (74777, 86453)	28292 (23395, 32857)
Heat capacity change (J/K mol-amino acid-residue), 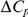	62.9 (61.9, 63.7)	68.9 (67.6, 70.3)	62.6 (61.7, 63.7)
Number of amino acid residues, 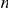	259.2 (232.9, 282.6)	211.8 (179.6, 252.8)	316.4 (290.8, 339.5)
Average number of non-polar hydrogen atoms per amino acid residue, 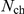	5.56 (5.49, 5.64)	6.06 (5.92, 6.25)	5.40 (5.32, 5.47)

On the right side axis of Figure 2 are shown the estimated posterior distributions of the parameters for each domain. The parameters 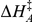, 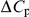, 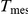 and 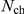 are largest in Archaea and least in Eukarya. By calculating the mean number of MCMC iterations in which each alternative ordering arises we obtain the most probable ordering for 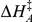: P(A>B>E) = 1.00; for 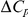: P(A>B>E) = 0.67; for 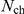: P(A>B>E) = 0.99; for 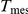: P (A>B>E) = 0.67. This is partly a result of the selection of strains used in this study because, apart from 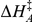
_,_ those parameters reflect the thermal stability of the RCR. The Eukarya strains included in this analysis are predominantly mesophilic, the Bacteria include more psychrophilic or psychrotrophic strains than the Archaea strains used, and the latter datasets include more thermophilic strains than the Bacteria, which included only two mildly thermophilic species, *S. thermophilus* and *Acidimicrobium ferrooxidans*, the former represented by multiple datasets (37–46).

The remaining parameter, 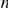, is largest in Eukarya, intermediate in Bacteria, and least in Archaea: P(E>B>A) = 0.98. Complete genome sequences give mean protein lengths of 449 for Eukarya, 330 for Bacteria, and 270 for Archaea [15]. With the most general assumptions our model is able to obtain the same ordering (Table 2).

For those species represented by at least three datasets we calculated the percentage deviation for the strain parameters (Table S3): 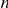 (37.5%), 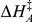 (23.6%), 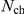 (5.7%), 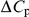 (1.9%), 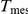 (0.5%). While the large variability for some parameters may be expected, the very low value for others such as 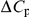 and 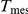 are of considerable interest. This can also be seen in Figure 2 where we use vertical shading to delimit species. The 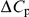 and 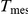 appear almost species-specific, as is, to a lesser extent, 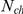. The parameter 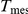 is mathematically related to 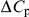 and so shares its low variability.

The 95% HPDI for the domain level 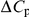 for Bacteria (61.9, 63.7) and Eukarya (61.7, 63.7) are contained within the range expected for mesophiles, 36.6–76.2 [16], and the posterior domain 95% HPDI for Archaea (67.6,70.3) is contained within the range for thermophiles: 56.7–118 [16]. The Archaea result is expected since the strains used in this study are mostly thermophiles. Estimates of 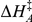 from microorganism growth [1], [17]–[23] have a very wide range, 34,601–110,000, which contains our 95% HPDI for Bacteria (64,887, 70,312) and Archaea (74,777, 86,453), but that for Eukarya is lower (23,395, 32,857). The low values for Eukarya stand in contrast to the domain ordering of 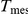, as the yeasts used here are predominantly mesophilic and, in the absence of a domain effect, would be expected to have activation energies intermediate between the bacterial and archaeal strains used here. The relatively low activation energies for Eukarya appear to suggest a genuine effect. They are very comparable to other estimates of activation energy for *Saccharomyces bayanus* var. *uvarum* [24] and conceivably may relate specifically to yeast species used in wine production, rather than to the Eukarya as a whole.

The quantity 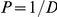, the probability that the enzyme is in its native (catalytically active) state [3], is shown in Figure 4 for each strain. Some strains have narrower curves indicating a more limited range of temperatures within which the protein is in the native state, but others are “flat-topped” indicating a more extensive range. As can be seen from the extrapolated curves the optimal stability region is fully observed for most strains. The squat curves exhibited by the strains that do not reach maximum probability suggest that those strains were not grown under optimal conditions.

As shown in Table 1, the 95% HPDI for 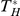 and 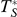 do not overlap, emphasizing that their values are not identical. Equivalently, the 95% HPDI for their difference, (−16, −13), does not include zero. In fact, they are quite close to the values suggested by others [8]–[10], 373 K and 385 K, respectively, and which are contained within their 95% HPDI.

## Discussion

Whereas previous work [3] individually analyzed 35 Bacteria strains and held 3 parameters fixed (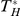, 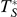, and 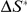) we were, with only minimal constraints, able to analyze all 95 Archaea, Bacteria, and Eukarya strains to provide estimates of all strain, domain and common parameters simultaneously, and obtain reasonable estimates of thermodynamic parameters previously estimated decades ago by experiment [9]–[10]. The model successfully described the growth of unicellular organisms in all domains of life. We were able to do this by utilizing the MCMC methods to greatly simplify the computation involved with a Bayesian framework, and a hierarchical model that permits those parameters that are less well informed to ‘borrow strength’ from elsewhere in the model and so improve estimation.

While it is possible that the reaction system is more complex and only appears equivalent to a single rate-controlling system, single enzymes have been shown to be growth rate-controlling [25]–[26]. The model results lend support to the ‘master reaction’ model in which an enzyme-catalyzed reaction limits growth rate at all temperatures. The model indicates that, all other things being equal, thermodynamic properties of protein hydration govern biological rates and these are consistent in all forms of unicellular life. In turn this suggests that the thermal stability of proteins is a fundamental property in the evolution and adaptation of life on earth and has importance for many fields of study including microbiology, protein chemistry, thermal biology, and ecological theory, including, for example, the influence of the vast microbial biomass and activity in the biosphere that is poorly described in current climate models [27].

Since this is only the debut of this model we anticipate further developments and extensions. Our results appear consistent with the assumption that growth is controlled by a single rate-limiting, enzyme-catalyzed ‘master reaction’ using an Arrhenius form modified by terms that describe the temperature-dependent denaturation of that enzyme. While there may be other explanations for our results, candidates for a single-enzyme rate-controlling mechanism common to all life can be conjectured. There may be processes that occur in parallel leading up to cell division, but it is also plausible that there is a single process dictated by a single enzyme (or other bio-catalyst) that could still be rate-limiting. There are various candidate biosynthetic processes common to all cells. Alternatively, anabolism or biosynthesis could be limited by the rate of energy generation e.g, via respiration. Regardless of whether there is a single rate-limiting, enzyme-catalyzed ‘master reaction’, or rate limiting process/pathway, there remains the possibility that some model parameters are more consequential at some temperatures than others. Whether the activation energy dominates the suboptimal range while denaturation is more important in the super optimal range is a topic for further investigation.

While we chose to adopt a model structure that recognises the three domains of life [4], the need for differentiation into three domain parameter sets remains to be explicitly considered. In the current study this was complicated by the availability of data. In particular, our data sets were not balanced in that they had disproportionate representation of thermal adaptation groups (mesophiles, thermophiles, and so on). Further work is needed to examine whether an alternative, and perhaps simpler, domain parameter structure may be as effective. In addition, there is a need to consider if there are systematic differences of thermodynamic parameters between thermal adaptation groups, either within or between domains.

The results of our current model indicate that the activation energy of the putative ‘master reaction’ varies among strains and probably among species. This is in contradiction to the ‘Metabolic Theory of Ecology’ (MTE) [28]–[29] which describes metabolism in terms of an Arrhenius temperature dependence and assumes the same activation energy for all organisms. Other recent work also supports the idea that activation energy is not invariant [30]–[31] or interprets the variation of activation energy as being the result of protein denaturation [32], as in this study.

We only considered the temperature dependent growth of unicellular poikilothermic organisms, but it may be possible to apply the model directly to simple multicellular organisms or the growth of anatomical structures [33]. For larger organisms an allometric power function of body mass could be used, analogous to that also assumed in the MTE [28]–[29], [34], whilst also remaining cognizant that the exponent in the function may not be the same for all forms of life [35]. While the current model well described temperature-dependent growth across the entire biokinetic range, practical application in environmental science is likely to require further extension to allow for additional factors such as nutrient and water availability and other environmental stressors. The extension to multicellularity and other environmental variables would broaden the model to a much wider ecological context. These would appear to be necessary steps before examining applications, e.g. in climate change, oceanography and soil carbon studies.

Many of these areas will have significant impact on aspirations of an improved human condition, especially for currently disadvantaged populations, balanced with the goal of maintaining biological diversity and activity in the long term. It has not escaped our attention that our work immediately suggests the possibility of temperature adaptation models applicable to wide ranging forms of life and life systems.

## Supporting Information

Figure S1 **Detailed fits for strains 1–16.** Shown are the observed growth rate data using symbols and fitted curves for strains 1–16. Observed data are shown as green circles. All are strains of Bacteria. The fitted curves are calculated using the mean posterior parameter estimates and extend beyond the observed temperature range by ±2.5°.
(TIF)

Figure S2 **Detailed fits for strains 17–32.** Shown are the observed growth rate data using symbols and fitted curves for strain 17–32. Observed data are shown as green circles. All are strains of Bacteria. The fitted curves are calculated using the mean posterior parameter estimates and extend beyond the observed temperature range by ±2.5°.
(TIF)

Figure S3 **Detailed fits for strains 33–48.** Shown are the observed growth rate data using symbols and fitted curves for strain 33–48. Observed data are shown as green circles for strains of Bacteria and blue squares for strains of Archaea. The fitted curves are calculated using the mean posterior parameter estimates and extend beyond the observed temperature range by ±2.5°.
(TIF)

Figure S4 **Detailed fits for strains 49–64.** Shown are the observed growth rate data using symbols and fitted curves for strain 49–64. Observed data are shown as blue squares for strains of Archaea. The fitted curves are calculated using the mean posterior parameter estimates and extend beyond the observed temperature range by ±2.5°.
(TIF)

Figure S5 **Detailed fits for strains 65–80.** Shown are the observed growth rate data using symbols and fitted curves for strain 65–80. Observed data are shown as blue squares for strains of Archaea and red diamonds for strains of Eukarya. The fitted curves are calculated using the mean posterior parameter estimates and extend beyond the observed temperature range by ±2.5°.
(TIF)

Figure S6 **Detailed fits for strains 81–95.** Shown are the observed growth rate data using symbols and fitted curves for strain 81–95. Observed data are shown as red diamonds. All are strains of Eukarya. The fitted curves are calculated using the mean posterior parameter estimates and extend beyond the observed temperature range by ±2.5°.
(TIF)

Table S1 **Estimates for the strain parameters.** Shown are the posterior means and subscripted standard deviations for each strain.
(DOC)

Table S2 **Priors for strain and domain parameters.**
(DOC)

Table S3 **Domain parameter percent deviation by species.** Shown are the maximum and minimum of the strain posterior means for each species, their difference, mid point, and percent deviation.
(DOC)

## References

[1] Johnson FH, Lewin I (1946). The growth rate of *E. coli* in relation to temperature, quinine and coenzyme.. J Cell Physiol.

[2] Murphy KP, Privalov PL, Gill SJ (1990). Common features of protein unfolding and dissolution of hydrophobic compounds.. Science.

[3] Ratkowsky DA, Olley J, Ross T (2005). Unifying temperature effects on the growth rate of bacteria and the stability of globular proteins.. J Theor Biol.

[4] Woese CR, Kandler O, Wheelis ML (1990). Towards a natural system of organisms: proposal for the domains Archaea, Bacteria, and Eucarya.. P Natl Acad Sci USA.

[5] Graziano G, Catanzano F, Barone G (1998). Prediction of the heat capacity change on thermal denaturation of globular proteins.. Thermochim Acta.

[6] Graziano G (2008). Is there a relationship between protein thermal stability and the denaturation heat capacity change?. J Therm Anal Calorim.

[7] Graziano G, Barone G (1996). Group additivity analysis of the heat capacity changes associated with the dissolution into water of different organic compounds.. J Am Chem Soc.

[8] Makhatadze GI, Privalov PL (1993). Contribution of hydration to protein-folding thermodynamics: I. The enthalpy of hydration.. J Mol Biol.

[9] Privalov PL, Gill SJ (1988). Stability of protein structure and hydrophobic interaction.. Adv Protein Chem.

[10] Privalov PL, Makhatadze GI (1993). Contribution of hydration to protein-folding thermodynamics: II. The entropy and Gibbs energy of hydration.. J Mol Biol.

[11] Brooks SP (1998). Markov chain Monte Carlo method and its application.. Statistician.

[12] Haario H, Saksman E, Tamminen J (2001). An adaptive Metropolis algorithm.. Bernoulli.

[13] Chib S, Greenberg E (1995). Understanding the Metropolis-Hastings algorithm.. J Am Stat Assoc.

[14] Gilks WR, Roberts GO, Gilks WR, Richardson S, Spiegelhalter DJ (1996). Strategies for improving MCMC.. Markov Chain Monte Carlo in Practice.

[15] Zhang J (2000). Protein-length distributions for the three domains of life.. Trends Genet.

[16] McCrary BS, Edmondson SP, Shriver JW (1996). Hyperthermophile protein folding thermodynamics: differential scanning calorimetry and chemical denaturation of Sac7d.. J Mol Biol.

[17] Billing E (1974). The effect of temperature on the growth of the fireblight pathogen, *Erwinia amylovora*.. J Appl Microbiol.

[18] Coultate TP, Sundaram TK (1975). Energetics of *Bacillus stearothermophilus* growth: molar growth yield and temperature effects on growth efficiency.. J Bacteriol.

[19] Hanu FJ, Morita RY (1962). Significance of temperature characteristic of growth.. J Bacteriol.

[20] Mennett RH, Nakayama TOM (1971). Influence of temperature on substrate and energy conversion in *Pseudomonas fluorescens*.. Appl Environ Microb.

[21] Ng H, Ingraham JL, Marr AG (1962). Damage and derepression in *Escherichia coli* resulting from growth at low temperatures.. J Bacteriol.

[22] Price PB, Sowers T (2004). Temperature dependence of metabolic rates for microbial growth, maintenance, and survival.. P Natl Acad Sci USA.

[23] Shaw MK (1967). Effect of abrupt temperature shift on the growth of mesophilic and psychrophilic yeasts.. J Bacteriol.

[24] Serra A, Strehaiano P, Taillandier P (2005). Influence of temperature and pH on *Saccharomyces bayanus* var. *uvarum* growth; impact of a wine yeast interspecific hybridization on these parameters.. Int J Food Microbiol.

[25] Ron EZ, Alajem S, Biran D, Grossman N (1990). Adaptation of *Escherichia coli* to elevated temperatures: the *metA* gene product is a heat shock protein.. Anton Leeuw Int G.

[26] Gur E, Biran D, Gazit E, Ron EZ (2002). *In vivo* aggregation of a single enzyme limits growth of *Escherichia coli* at elevated temperatures.. Mol Microbiol.

[27] Anonymous (2008). http://genomicscience.energy.gov/carboncycle/report/.

[28] Gillooly JF, Brown JH, West GB, Savage VM, Charnov EL (2001). Effects of size and temperature on metabolic rate.. Science.

[29] Gillooly JF, Charnov EL, West GB, Savage VM, Brown JH (2002). Effects of size and temperature on development time.. Nature.

[30] Clarke A, Fraser KPP (2004). Whey does metabolism scale with temperature?. Funct Ecol.

[31] Knies JL, Kingsolver JG (2010). Erroneous Arrhenius: Modified Arrhenius model best explains the temperature dependence of ectotherm fitness.. Am Nat.

[32] Dell AI, Pawar S, Savage VM (2011). Systematic variation in the temperature dependence of physiological and ecological traits.. P Natl Acad Sci USA.

[33] McMeekin TA, Olley JN, Ross T, Ratkowsky DA (1993).

[34] Savage VM, Gillooly JF, Brown JH, West GB, Charnov EL (2004). Effects of body size and temperature on population growth.. Am Nat.

[35] Isaac NJB, Carbone C (2010). Why are metabolic scaling exponents so controversial? Quantifying variance and testing hypotheses.. Ecol Lett.

[36] Ingraham JL (1958). Growth of psychrophilic bacteria.. J Bacteriol.

[37] Raison JK (1973). The Influence of temperature-induced phase changes on the kinetics of respiratory and other membrane-associated enzyme systems.. J Bioenerg Biomembr.

[38] Raison JK (1973). Temperature-induced phase changes in membrane lipids and their influence in metabolic regulation..

[39] Ragone R (2004). Phenomenological similarities between protein denaturation and small-molecule dissolution: Insights into the mechanism driving the thermal resistance of globular proteins.. Proteins.

[40] Franks F (1988). Description and Classification of Proteins.

[41] Honda S, Yamasaki K, Sawada Y, Morii H (2004). 10 Residue folded peptide designed by segment statistics.. Structure.

[42] Kimball J (2006). Proteins.. http://users.rcn.com/jkimball.ma.ultranet/BiologyPages/P/Proteins.html.

[43] Liu L, Yang C, Guo Q-X (2000). A study on the enthalpy–entropy compensation in protein unfolding.. Biophys Chem.

[44] Jiang X, Farid H, Pistor E, Farid RS (2000). A new approach to the design of uniquely folded thermally stable proteins.. Protein Sci.

