# Supplementary figures and images for: Universality of Thermodynamic Constants Governing Biological Growth Rates
Source: PLoS One. 2012 Feb 14;7(2):e32003. doi: 10.1371/journal.pone.0032003 (PMC3279425; doi:10.1371/journal.pone.0032003)

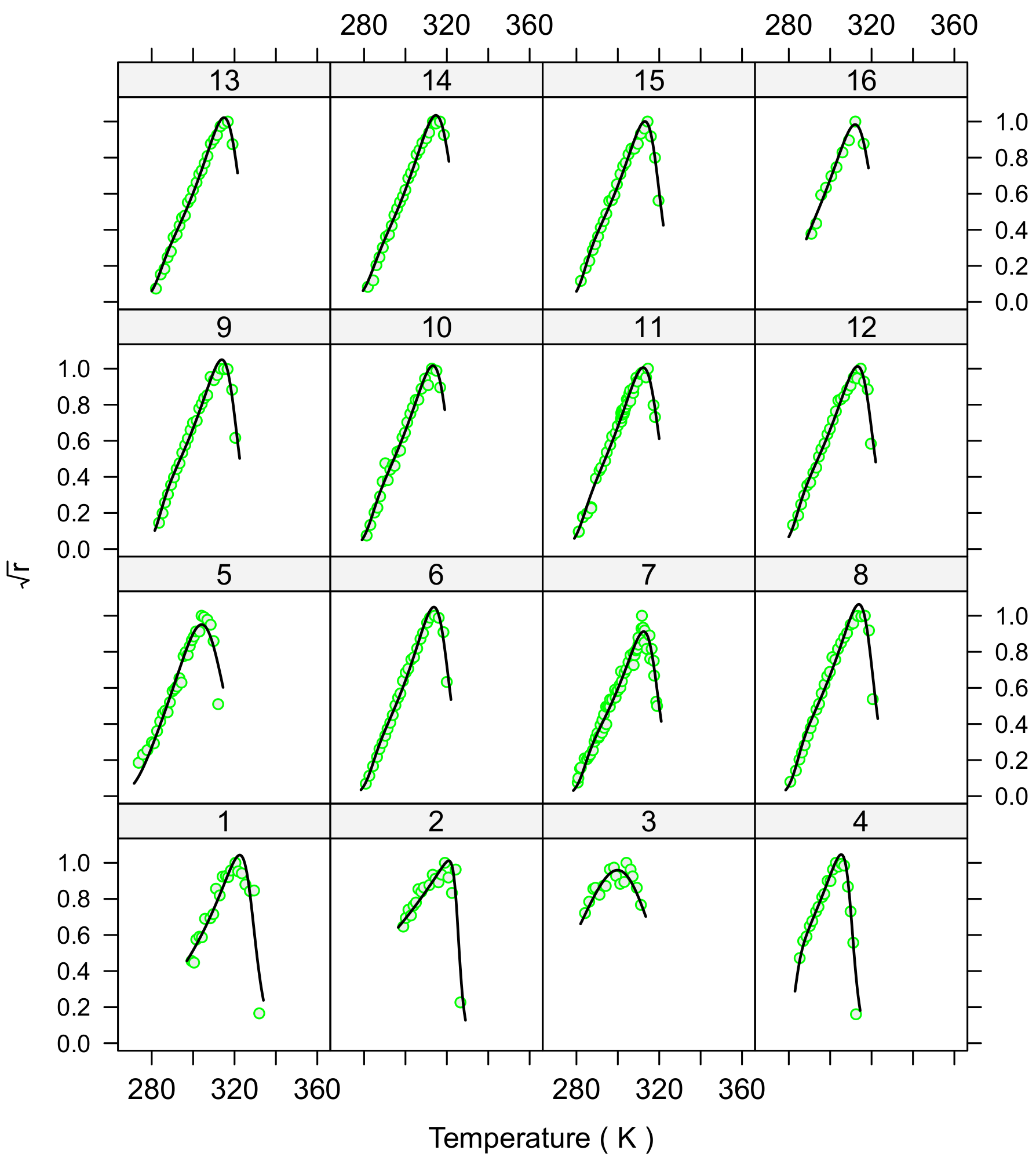

Supplement: Figure S1 — Detailed fits for strains 1–16. Shown are the observed growth rate data using symbols and fitted curves for strains 1–16. Observed data are shown as green circles. All are strains of Bacteria. The fitted curves are calculated using the mean posterior parameter estimates and extend beyond the observed temperature range by ±2.5°. (TIF) [file pone.0032003.s001.tif]

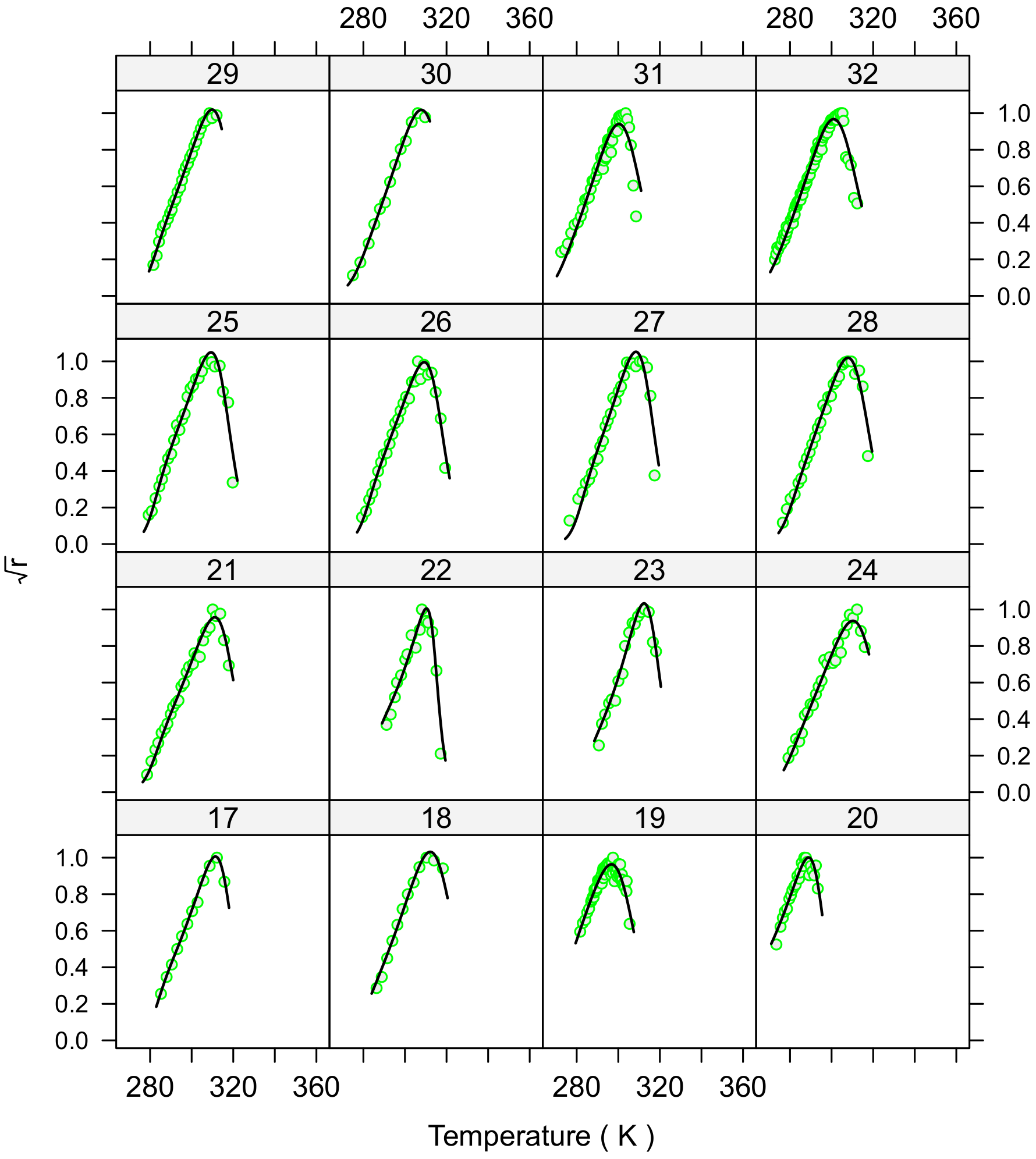

Supplement: Figure S2 — Detailed fits for strains 17–32. Shown are the observed growth rate data using symbols and fitted curves for strain 17–32. Observed data are shown as green circles. All are strains of Bacteria. The fitted curves are calculated using the mean posterior parameter estimates and extend beyond the observed temperature range by ±2.5°. (TIF) [file pone.0032003.s002.tif]

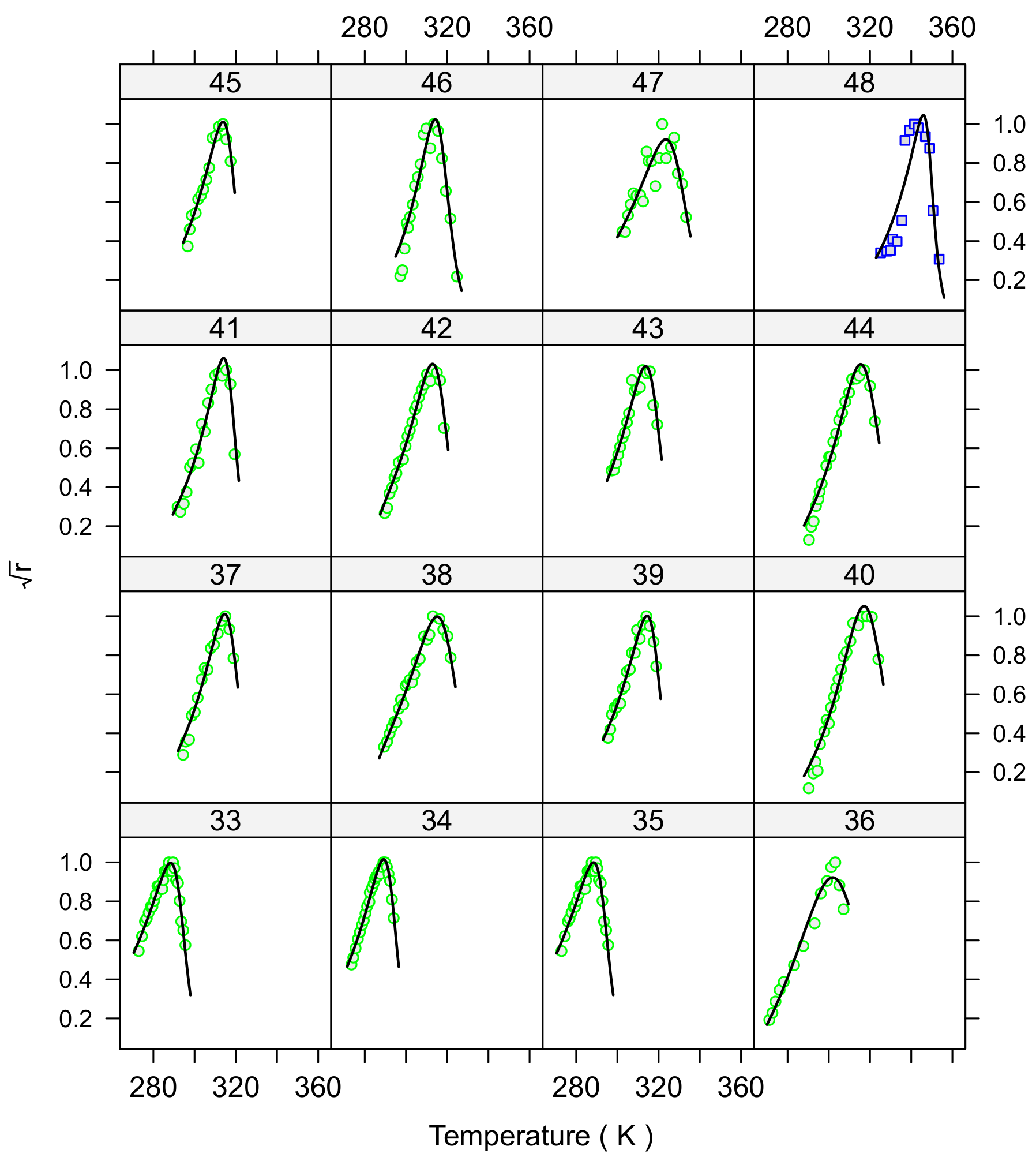

Supplement: Figure S3 — Detailed fits for strains 33–48. Shown are the observed growth rate data using symbols and fitted curves for strain 33–48. Observed data are shown as green circles for strains of Bacteria and blue squares for strains of Archaea. The fitted curves are calculated using the mean posterior parameter estimates and extend beyond the observed temperature range by ±2.5°. (TIF) [file pone.0032003.s003.tif]

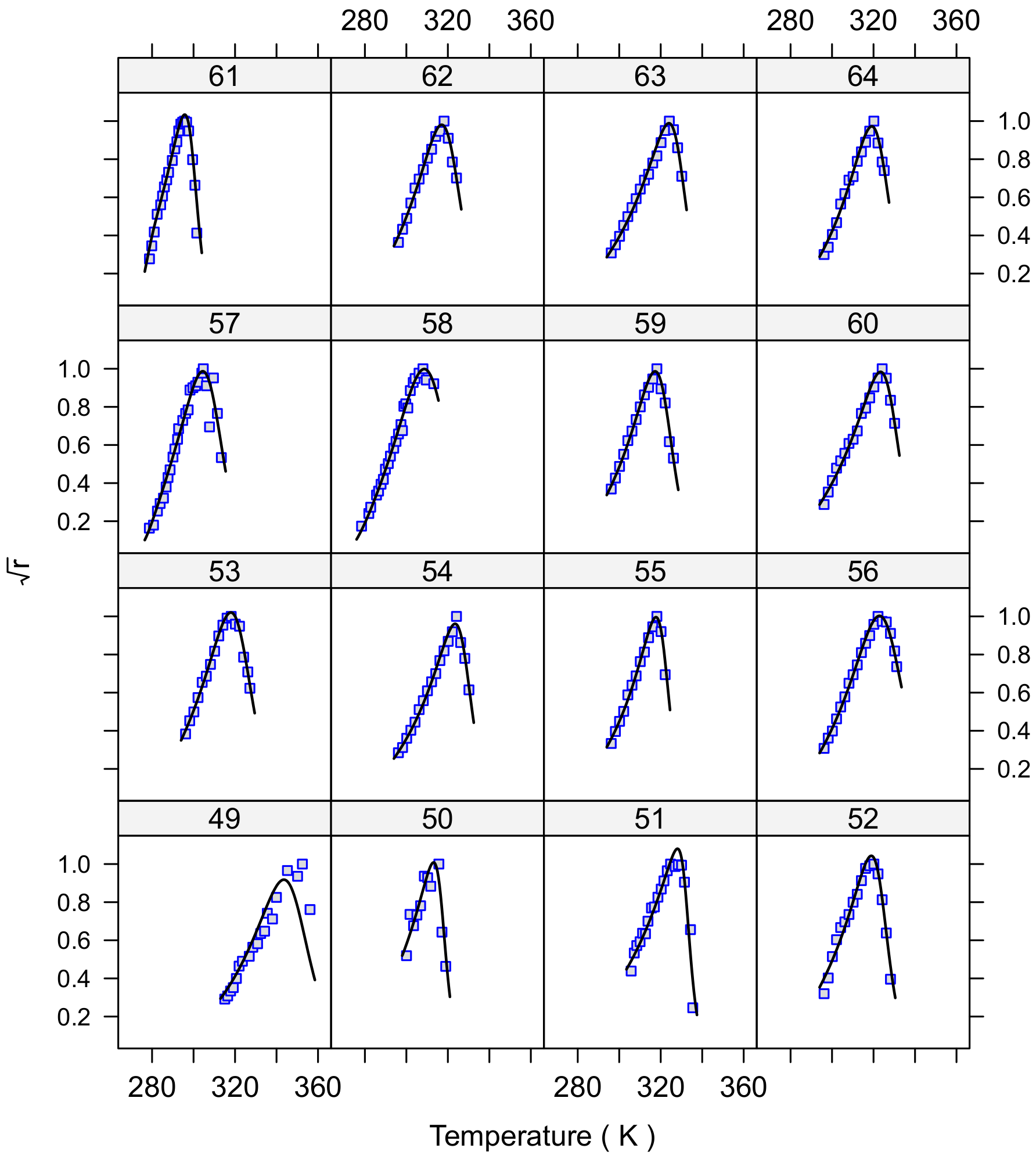

Supplement: Figure S4 — Detailed fits for strains 49–64. Shown are the observed growth rate data using symbols and fitted curves for strain 49–64. Observed data are shown as blue squares for strains of Archaea. The fitted curves are calculated using the mean posterior parameter estimates and extend beyond the observed temperature range by ±2.5°. (TIF) [file pone.0032003.s004.tif]

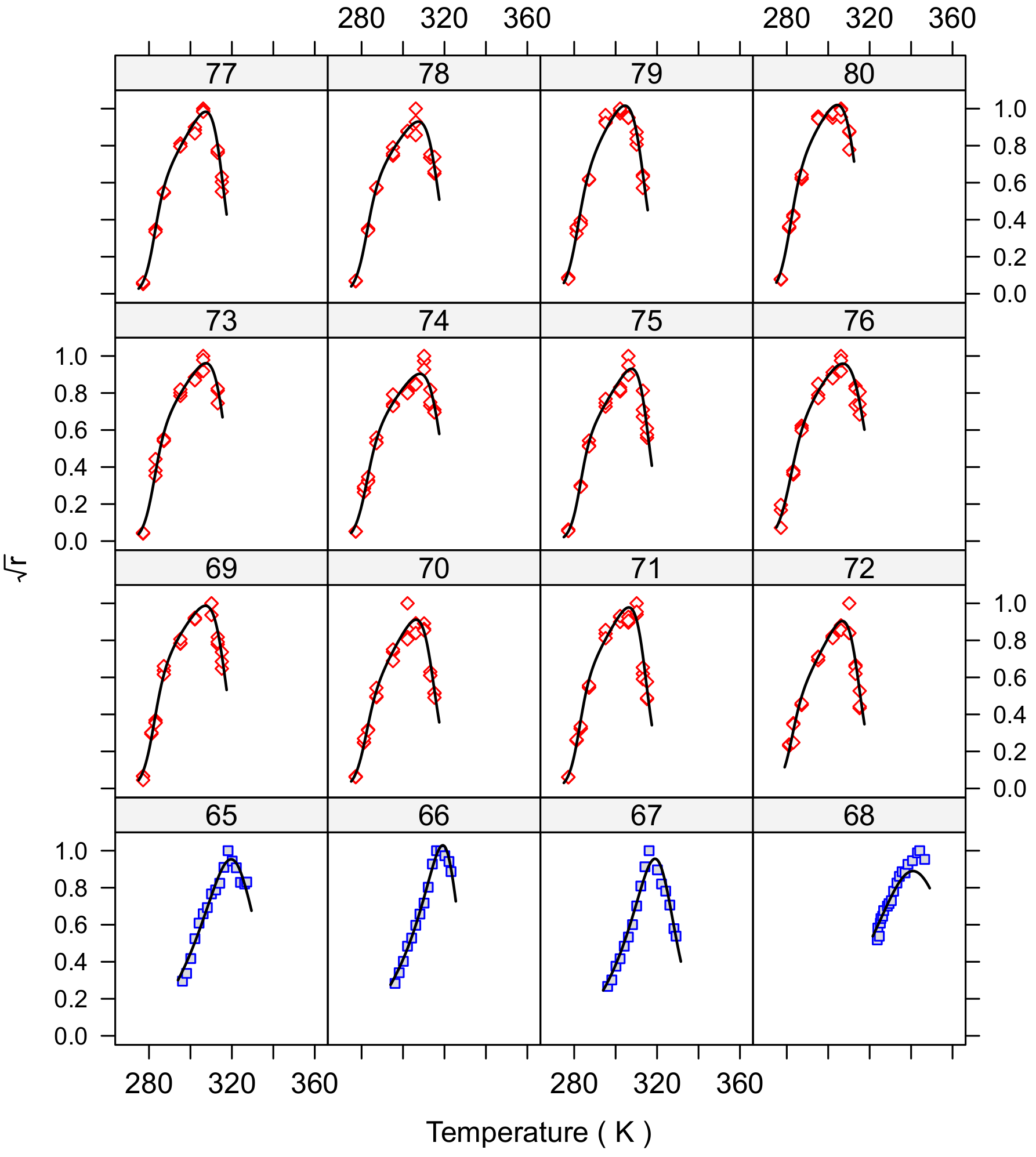

Supplement: Figure S5 — Detailed fits for strains 65–80. Shown are the observed growth rate data using symbols and fitted curves for strain 65–80. Observed data are shown as blue squares for strains of Archaea and red diamonds for strains of Eukarya. The fitted curves are calculated using the mean posterior parameter estimates and extend beyond the observed temperature range by ±2.5°. (TIF) [file pone.0032003.s005.tif]

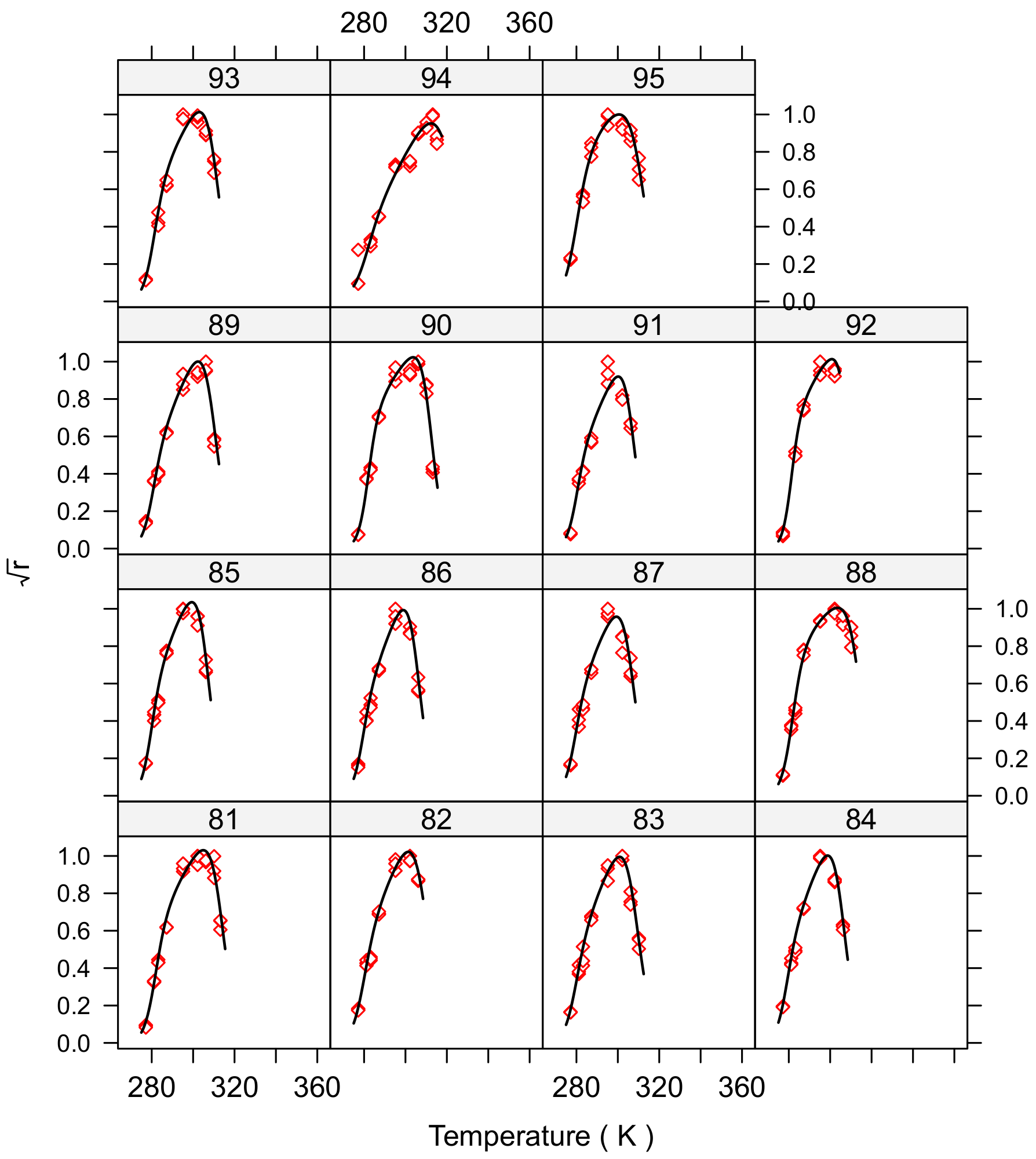

Supplement: Figure S6 — Detailed fits for strains 81–95. Shown are the observed growth rate data using symbols and fitted curves for strain 81–95. Observed data are shown as red diamonds. All are strains of Eukarya. The fitted curves are calculated using the mean posterior parameter estimates and extend beyond the observed temperature range by ±2.5°. (TIF) [file pone.0032003.s006.tif]
