# Supplementary material for: Universality of Thermodynamic Constants Governing Biological Growth Rates
Source: PLoS One. 2012 Feb 14;7(2):e32003. doi: 10.1371/journal.pone.0032003 (PMC3279425; doi:10.1371/journal.pone.0032003)
Supplement: Table S1 — Estimates for the strain parameters. Shown are the posterior means and subscripted standard deviations for each strain. (DOC) [file pone.0032003.s007.doc]

**Table S1. Estimates for the strain parameters.**

**Shown are the posterior means and subscripted standard deviations for each strain.**

| Strain ID | Domain | Strain name | Source of the data | Enthalpy of activation  (J/mol), $\Delta H_{A}^{\text{‡}}$ | Heat capacity change  (J/K mol-amino acid-residue), $\Delta C_{p}$ | Number of amino acid residues, $n$ | Average number of non-polar hydrogen atoms per amino acid residue, $N_{\text{ch}}$ |
| --- | --- | --- | --- | --- | --- | --- | --- |
| 1 | Bacteria | *Acidimicrobium ferrooxidans* | [1] | 54527_6253_ | 70.9_0.54_ | 261_49.2_ | 6.0_0.120_ |
| 2 | Bacteria | *Acidithiobacillus caldus* | [1] | 28438_4158_ | 70.0_0.25_ | 569_111.2_ | 5.4_0.104_ |
| 3 | Bacteria | *Acidithiobacillus ferrooxidans* | [1] | 47754_8008_ | 52.5_2.91_ | 80_13.7_ | 6.3_0.163_ |
| 4 | Bacteria | *Acidithiobacillus thiooxidans* | [1] | 49433_3624_ | 62.5_0.19_ | 605_52.6_ | 5.0_0.038_ |
| 5 | Bacteria | *Aeromonas hydrophila* (3459) | [2] | 72404_4739_ | 59.1_0.64_ | 173_12.3_ | 5.7_0.048_ |
| 6 | Bacteria | *Escherichia coli* (0111.H-) | [3] | 70436_2881_ | 66.1_0.29_ | 282_29.8_ | 5.7_0.070_ |
| 7 | Bacteria | *Escherichia coli* (M23) | [3] | 67555_2494_ | 65.6_0.20_ | 313_25.3_ | 5.6_0.052_ |
| 8 | Bacteria | *Escherichia coli* (NT.R31) | [3] | 63511_2952_ | 66.3_0.30_ | 303_33.8_ | 5.6_0.072_ |
| 9 | Bacteria | *Escherichia coli* (O126.H21) | [3] | 62925_3275_ | 66.3_0.36_ | 286_36.7_ | 5.7_0.087_ |
| 10 | Bacteria | *Escherichia coli* (O157.H7) | [3] | 66188_2995_ | 65.9_0.38_ | 269_39.5_ | 5.7_0.108_ |
| 11 | Bacteria | *Escherichia coli* (O157.H-) | [3] | 67060_2853_ | 65.3_0.27_ | 263_24.5_ | 5.7_0.064_ |
| 12 | Bacteria | *Escherichia coli* (O81.H.R106) | [3] | 63351_3115_ | 66.0_0.33_ | 284_34.9_ | 5.7_0.081_ |
| 13 | Bacteria | *Escherichia coli* (O88.H-.R171) | [3] | 69449_3411_ | 66.6_0.49_ | 249_39.9_ | 5.8_0.125_ |
| 14 | Bacteria | *Escherichia coli* (O88.H-.R172) | [3] | 70029_3552_ | 66.4_0.48_ | 227_33.8_ | 5.9_0.116_ |
| 15 | Bacteria | *Escherichia coli* (ONT.H8.R91) | [3] | 65861_2951_ | 65.9_0.28_ | 305_32.1_ | 5.6_0.067_ |
| 16 | Bacteria | *Escherichia coli* (SB1.0.977B) | [4] | 66935_4018_ | 65.1_0.65_ | 233_49.9_ | 5.8_0.159_ |
| 17 | Bacteria | *Escherichia coli* (SB1.0.998B) | [4] | 66001_3580_ | 64.9_0.57_ | 256_48.8_ | 5.7_0.139_ |
| 18 | Bacteria | *Escherichia coli* (SB1.0.998) | [4] | 69248_5246_ | 63.9_1.25_ | 153_29.0_ | 6.1_0.141_ |
| 19 | Bacteria | *Gelidibacter* sp. (IC158) | [5] | 53764_10810_ | 55.6_1.88_ | 188_40.4_ | 5.5_0.111_ |
| 20 | Bacteria | *Glaciecola punicea* (ACAM 611) | [5] | 53749_6034_ | 49.1_2.07_ | 291_71.6_ | 4.8_0.111_ |
| 21 | Bacteria | *Klebsiella oxytoca* (NRRL.B-199) | [6] | 61048_3366_ | 64.6_0.50_ | 205_22.1_ | 5.9_0.080_ |
| 22 | Bacteria | *Leptospirillum ferrooxidans* | [1] | 69875_3807_ | 64.5_0.18_ | 513_44.3_ | 5.2_0.041_ |
| 23 | Bacteria | *Leptospirillum ferriphilum* | [1] | 85067_3955_ | 65.0_0.36_ | 264_27.2_ | 5.7_0.068_ |
| 24 | Bacteria | *Listeria monocytogenes* (Run 1) | [7] | 59369_4942_ | 63.2_1.08_ | 149_20.1_ | 6.1_0.107_ |
| 25 | Bacteria | *Listeria monocytogenes* (Run 2) | [7] | 57617_3539_ | 64.0_0.38_ | 242_16.4_ | 5.7_0.041_ |
| 26 | Bacteria | *Listeria monocytogenes* (Run 3) | [7] | 59772_3568_ | 63.9_0.40_ | 239_18.1_ | 5.7_0.046_ |
| 27 | Bacteria | *Listeria monocytogenes* (Run 4) | [7] | 63582_3546_ | 63.4_0.40_ | 256_21.8_ | 5.6_0.050_ |
| 28 | Bacteria | *Listeria monocytogenes* (Run 5) | [7] | 62489_3722_ | 62.6_0.47_ | 199_13.2_ | 5.8_0.042_ |
| 29 | Bacteria | *Listeria monocytogenes* (Scott A) | [8] | 63124_4198_ | 63.8_0.71_ | 209_32.9_ | 5.8_0.123_ |
| 30 | Bacteria | *Paracoccus halodenitrificans* (0.964) | [4] | 66346_4078_ | 61.7_0.66_ | 162_15.8_ | 5.9_0.091_ |
| 31 | Bacteria | *Pseudomonas fluorescens* (1412) | [9] | 70296_6068_ | 56.9_0.81_ | 182_18.1_ | 5.6_0.063_ |
| 32 | Bacteria | *Pseudomonas putida* (1442) | [9] | 69349_5394_ | 56.7_0.85_ | 159_10.8_ | 5.7_0.045_ |
| 33 | Bacteria | *Shewanella gelidimarina* (1.0pct) | [10] | 50451_6192_ | 48.1_2.18_ | 337_81.5_ | 4.7_0.070_ |
| 34 | Bacteria | *Shewanella gelidimarina* (2.5pct) | [10] | 64437_5877_ | 48.7_2.01_ | 377_94.3_ | 4.6_0.083_ |
| 35 | Bacteria | *Shewanella gelidimarina* (4.0pct) | [10] | 50795_5642_ | 48.3_1.98_ | 343_77.4_ | 4.7_0.066_ |
| 36 | Bacteria | *Spirillum* (L9) | [11] | 65767_5501_ | 55.6_1.02_ | 121_11.5_ | 5.9_0.094_ |
| 37 | Bacteria | *Streptococcus thermophilus* (BC-14-1) | [12] | 86769_4003_ | 66.2_0.29_ | 300_36.8_ | 5.6_0.084_ |
| 38 | Bacteria | *Streptococcus thermophilus* (BC-14-2) | [12] | 66002_3963_ | 66.5_0.79_ | 189_34.1_ | 6.1_0.141_ |
| 39 | Bacteria | *Streptococcus thermophilus* (BC-14-3) | [12] | 78548_4053_ | 66.3_0.25_ | 328_42.2_ | 5.6_0.084_ |
| 40 | Bacteria | *Streptococcus thermophilus* (BC-14-5) | [12] | 97226_4160_ | 66.2_0.51_ | 171_13.2_ | 6.1_0.059_ |
| 41 | Bacteria | *Streptococcus thermophilus* (BC-29-1) | [12] | 89340_3846_ | 66.0_0.21_ | 381_35.9_ | 5.4_0.054_ |
| 42 | Bacteria | *Streptococcus thermophilus* (BC-29-2) | [12] | 80039_3687_ | 65.4_0.36_ | 279_32.2_ | 5.6_0.078_ |
| 43 | Bacteria | *Streptococcus thermophilus* (BC-29-3) | [12] | 80798_4308_ | 65.9_0.28_ | 291_27.8_ | 5.6_0.062_ |
| 44 | Bacteria | *Streptococcus thermophilus* (BC-29-5) | [12] | 93964_4316_ | 65.7_0.52_ | 190_16.7_ | 6.0_0.063_ |
| 45 | Bacteria | *Streptococcus thermophilus* (K118-4) | [12] | 83867_4409_ | 66.0_0.25_ | 352_47.6_ | 5.5_0.086_ |
| 46 | Bacteria | *Streptococcus thermophilus* (K120-6) | [12] | 106477_4122_ | 65.7_0.19_ | 324_15.8_ | 5.5_0.029_ |
| 47 | Bacteria | *Sulfobacillus thermosulfidooxidans* | [1] | 62572_6513_ | 70.9_0.92_ | 154_25.1_ | 6.5_0.139_ |
| 48 | Archaea | *Acidianus brierleyi* | [1] | 101934_8827_ | 83.2_0.69_ | 254_76.5_ | 6.7_0.183_ |
| 49 | Archaea | *Acidianus brierleyi* (sulphur) | [1] | 77512_12080_ | 81.5_5.64_ | 88_78.6_ | 8.6_0.914_ |
| 50 | Archaea | *Ferroplasma acidiphilum* | [1] | 76780_5131_ | 65.8_0.22_ | 446_51.1_ | 5.3_0.061_ |
| 51 | Archaea | *Ferroplasma cyprexacervatum* | [1] | 61574_7539_ | 73.5_0.52_ | 318_114.8_ | 6.0_0.152_ |
| 52 | Archaea | *Haloarcula vallismortis* | [13] | 73397_4677_ | 68.6_0.39_ | 243_23.5_ | 5.9_0.066_ |
| 53 | Archaea | *Halobaculum gomorrense* | [13] | 79969_5187_ | 67.3_0.61_ | 170_14.6_ | 6.2_0.064_ |
| 54 | Archaea | *Halococcus morrhuae* | [13] | 75559_4485_ | 70.8_0.45_ | 221_33.2_ | 6.1_0.123_ |
| 55 | Archaea | *Haloferax volcanii* | [13] | 80970_4322_ | 67.9_0.28_ | 330_48.3_ | 5.6_0.098_ |
| 56 | Archaea | *Halogeometricum borinquense* | [13] | 78593_5264_ | 69.3_0.94_ | 128_15.7_ | 6.6_0.111_ |
| 57 | Archaea | *Halorubrum lacusprofundi* (ACAM 32) | [14] | 81634_6825_ | 60.2_0.72_ | 235_20.4_ | 5.5_0.043_ |
| 58 | Archaea | *Halorubrum lacusprofundi* (ACAM 34) | [14] | 78293_4757_ | 61.1_0.86_ | 152_15.4_ | 6.0_0.076_ |
| 59 | Archaea | *Halorubrum saccharovorum* | [13] | 79889_4848_ | 67.5_0.42_ | 223_19.0_ | 5.9_0.060_ |
| 60 | Archaea | *Haloterrigena turkmenica* | [13] | 70975_5590_ | 70.8_0.88_ | 184_36.2_ | 6.3_0.182_ |
| 61 | Archaea | *Methanococcoides burtonii* | [15] | 82982_7851_ | 56.5_0.52_ | 588_84.6_ | 4.7_0.052_ |
| 62 | Archaea | *Natrialba asiatica* | [13] | 80316_4967_ | 67.1_0.58_ | 198_25.3_ | 6.0_0.093_ |
| 63 | Archaea | *Natrinema pellirubrum* | [13] | 69268_4846_ | 71.2_0.65_ | 205_42.4_ | 6.2_0.184_ |
| 64 | Archaea | *Natronobacterium gregoryi* | [13] | 83873_5092_ | 68.2_0.62_ | 206_29.9_ | 6.1_0.115_ |
| 65 | Archaea | *Natronococcus occultus* | [13] | 86492_5256_ | 66.5_1.03_ | 121_14.4_ | 6.6_0.101_ |
| 66 | Archaea | *Natronomonas bangense* | [13] | 90109_4875_ | 68.2_0.47_ | 227_35.4_ | 6.0_0.127_ |
| 67 | Archaea | *Natronomonas pharaonis* | [13] | 98693_4906_ | 67.2_0.43_ | 171_9.4_ | 6.2_0.042_ |
| 68 | Archaea | *Sulfolobus metallicus* | [1] | 85192_10187_ | 75.2_4.36_ | 54_31.6_ | 9.0_0.788_ |
| 69 | Eukarya | *Sacchararomyces cerevisiae* CECT 10131 | [16] | 25050_2988_ | 64.5_0.22_ | 279_16.7_ | 5.6_0.042_ |
| 70 | Eukarya | *Sacchararomyces cerevisiae* T73 | [16] | 35371_3390_ | 63.5_0.24_ | 300_17.2_ | 5.5_0.035_ |
| 71 | Eukarya | *Sacchararomyces cerevisiae* PE35 M | [16] | 31484_2914_ | 63.7_0.18_ | 337_18.6_ | 5.4_0.033_ |
| 72 | Eukarya | *Sacchararomyces cerevisiae* CPE7 | [16] | 38668_3161_ | 63.6_0.21_ | 320_20.8_ | 5.4_0.039_ |
| 73 | Eukarya | *Sacchararomyces cerevisiae* KYOKAI CBS 6412 | [16] | 26910_3056_ | 64.5_0.24_ | 290_21.3_ | 5.6_0.054_ |
| 74 | Eukarya | *Sacchararomyces cerevisiae* TEMOHAYA | [16] | 25458_2649_ | 65.0_0.23_ | 257_15.8_ | 5.7_0.047_ |
| 75 | Eukarya | *Sacchararomyces cerevisiae* Qa23 | [16] | 31714_2795_ | 64.3_0.18_ | 340_25.9_ | 5.4_0.047_ |
| 76 | Eukarya | *Sacchararomyces cerevisiae* TTA | [16] | 25533_2674_ | 64.5_0.26_ | 224_13.9_ | 5.8_0.048_ |
| 77 | Eukarya | *Sacchararomyces cerevisiae* PDM | [16] | 31441_2774_ | 64.2_0.19_ | 325_21.6_ | 5.5_0.041_ |
| 78 | Eukarya | *Sacchararomyces cerevisiae* RVA | [16] | 25709_3010_ | 64.6_0.23_ | 281_19.2_ | 5.6_0.047_ |
| 79 | Eukarya | *Sacchararomyces paradoxus* (CECT 1939*)* | [16] | 29604_3399_ | 62.9_0.24_ | 302_14.5_ | 5.4_0.029_ |
| 80 | Eukarya | *Sacchararomyces paradoxus* (120M) | [16] | 26840_3554_ | 63.0_0.28_ | 300_16.4_ | 5.5_0.040_ |
| 81 | Eukarya | *Sacchararomyces paradoxus* (K54) | [16] | 27510_3059_ | 63.2_0.21_ | 300_16.1_ | 5.5_0.035_ |
| 82 | Eukarya | *Sacchararomyces bayanus* var. uvarum (NCAIM 789) | [16] | 35753_4435_ | 60.9_0.34_ | 298_17.7_ | 5.4_0.046_ |
| 83 | Eukarya | *Sacchararomyces bayanus* var. *uvarum* (BM58) | [16] | 39527_4948_ | 60.5_0.33_ | 320_15.3_ | 5.3_0.026_ |
| 84 | Eukarya | *Sacchararomyces kudriavzevii* (CA111) | [16] | 40967_6508_ | 59.4_0.38_ | 381_25.5_ | 5.1_0.036_ |
| 85 | Eukarya | *Sacchararomyces kudriavzevii* (CR85) | [16] | 36128_5845_ | 59.9_0.33_ | 395_25.3_ | 5.1_0.035_ |
| 86 | Eukarya | *Sacchararomyces kudriavzevii* (CR89) | [16] | 44323_6985_ | 59.4_0.38_ | 407_31.9_ | 5.1_0.040_ |
| 87 | Eukarya | *Sacchararomyces kudriavzevii* (CR90) | [16] | 38124_7059_ | 59.8_0.44_ | 362_27.1_ | 5.2_0.041_ |
| 88 | Eukarya | *Sacchararomyces mikatae* (NBRC 1815) | [16] | 15203_2397_ | 63.4_0.22_ | 306_17.1_ | 5.5_0.044_ |
| 89 | Eukarya | *Sacchararomyces arboricolus* (CBS 10644) | [16] | 39121_4154_ | 61.4_0.25_ | 333_18.0_ | 5.3_0.032_ |
| 90 | Eukarya | *Sacchararomyces cariocanus* (CBS 8841) | [16] | 22282_3162_ | 62.9_0.18_ | 372_19.3_ | 5.3_0.030_ |
| 91 | Eukarya | *Hanseniaspora uvarum* (CECT 10389) | [16] | 40853_5928_ | 60.2_0.32_ | 408_24.7_ | 5.1_0.034_ |
| 92 | Eukarya | *Candida stellata* (CECT 11108) | [16] | 25442_5625_ | 61.1_0.59_ | 475_54.1_ | 5.1_0.085_ |
| 93 | Eukarya | *Torulaspora delbrueckii* | [16] | 30412_3637_ | 62.1_0.27_ | 323_17.8_ | 5.4_0.032_ |
| 94 | Eukarya | *Kluyveromyces marxianus* | [16] | 34577_3162_ | 66.3_0.60_ | 153_14.0_ | 6.2_0.103_ |
| 95 | Eukarya | *Pichia fermentans* | [16] | 16348_2788_ | 61.9_0.25_ | 260_11.0_ | 5.5_0.032_ |

**References to data sources in Table S1**

1. Franzmann PD, Haddad CM, Hawkes RB, Robertson WJ, Plumb JJ (2005) Effects of temperature on the rates of iron and sulfur oxidation by selected bioleaching Bacteria and Archaea: Application of the Ratkowsky equation. Miner Eng 18: 1304-1314.

2. Hayward LJ (1990) Predictive microbiology of Aeromonas hydrophila: the effect of temperature and water activity on the growth of *Aeromonas hydrophila* [B.Sc. (Honours)]. Hobart: University of Tasmania.

3. Salter MA, Ross T, McMeekin TA (1998) Applicability of a model for non-pathogenic *Escherichia coli* for predicting the growth of pathogenic *Escherichia coli*. J Appl Microbiol 85: 357-364.

4. Krist K (1997) Descriptions and mechanisms of bacterial growth responses to water activity and compatible solutes [PhD]. Hobart: University of Tasmania.

5. Nichols DS, Greenhill AR, Shadbolt CT, Ross T, McMeekin TA (1999) Physicochemical parameters for the growth of sea ice bacteria *Glaciecola punicea* ACAM 611T and *Gelidibacter* sp. Strain IC158. Appl Environ Microb 65: 3757-3760.

6. Mellefont L (2000) Predictive model development and lag phase characterisation for applications in the meat industry [PhD]. Hobart: University of Tasmania.

7. Nichols DS, Presser KA, Olley J, Ross T, McMeekin T (2002) Variation of branched-chain fatty acids marks the normal physiological range for growth in *Listeria monocytogenes*. Appl Environ Microb 66: 2809-2813.

8. Ross T (1993) A Philosophy for the Development of Kinetic Models in Predictive Microbiology [PhD]. Hobart: University of Tasmania.

9. Neumeyer K (1995) Modelling pseudomonad growth in milk and milk-based products [Master's]. Hobart: University of Tasmania.

10. Nichols DS, Olley J, Garda J, Brenner RR, McMeekin T (2000) Effect of temperature and salinity stress on growth and lipid composition of *Shewanella gelidmarina*. Appl Environ Microb 66: 2422-2429.

11. Harder W, Veldkamp H (1971) Competition of marine psychrophilic bacteria at low temperatures. Anton Leeuw Int G 37: 51-63.

12. Benson JG (1996) Biofilms in pasteurisers: investigating growth of *Streptococcus thermophilus* [B. Sc. (Honours)]. Hobart: University of Tasmania.

13. Robinson JL, Pyzyna B, Atrasz RG, Henderson CA, Morrill KL, et al. (2005) Growth kinetics of extremely halophilic Archaea (family Halobacteriaceae) as revealed by Arrhenius plots. J Bacteriol 187: 923-929.

14. McMeekin TA, Franzmann PD (1988) Effect of temperature on the growth rates of halotolerant and halophilic Bacteria isolated from Antarctic saline lakes. Polar Biol 8: 281-285.

15. Franzmann PD, Springer N, Ludwig W, Conway de Macario E, Rohde M (1992) A methanogenic archaeon from Ace Lake, Antarctica: *Methanococcoides burtonii* sp. nov. Syst Appl Microbiol 15: 573-581.

16. Salvadó Z, Arroyo-López FN, Guillamón JM, Salazar G, Querol A, et al. (2011) Temperature adaptation markedly determines evolution within the *Saccharomyces* genus. Appl Environ Microb: 2292-2302.
