# Supplementary material for: Universality of Thermodynamic Constants Governing Biological Growth Rates
Source: PLoS One. 2012 Feb 14;7(2):e32003. doi: 10.1371/journal.pone.0032003 (PMC3279425; doi:10.1371/journal.pone.0032003)
Supplement: Table S2 — Priors for strain and domain parameters. (DOC) [file pone.0032003.s008.doc]

## Tables S2. Priors for strain and domain parameters.

| **Parameter [[1]](#footnote-2)** | **Priors[[2]](#footnote-3)** |
| --- | --- |
| Scaling constant | $c_{j}\sim\text{N}\left(c_{d\left(j\right)},\exp\left(\tau_{c}\right)\right)\left[-2,40\right]$ |
|  | $c_{d}\sim\text{Unif}\left(-2,40\right)$ |
|  | $\tau_{c}\sim\text{Unif}\left(-6.4537,-0.9085\right)$ |
| Enthalpy of activation [1,17-23,36-38] |  |
|  |  |
|  | $\tau_{\Delta H_{A}^{\text{‡}}}\sim\text{Unif}\left(-21.9926,-16.4474\right)$ |
| Heat capacity change [16,39] |  |
|  |  |
|  | $\tau_{\Delta C_{\text{p}}}\sim\text{Unif}\left(-6.8289,-1.2837\right)$ |
| Number of amino acid residues [40-42] | $n_{\text{j}}\sim\text{N}\left(n_{\text{d}\left(\text{j}\right)},\exp\left(\tau_{n}\right)\right)\left[10,20000\right]$ |
|  | $n_{d}\sim\text{Unif}\left(10,20000\right)$ |
|  | $\tau_{n}\sim\text{Unif}\left(-13.3692,-7.8240\right)$ |
| Enthalpy change at convergence temperature [43] | $\Delta H^{*}\sim\text{Unif}\left(3000,7000\right)$ |
| Entropy change at convergence temperature [43] | $\Delta S^{*}\sim\text{Unif}\left(10,30\right)$ |
| Convergence temperature for enthalpy [39,43-44] | $T_{H}\sim\text{Unif}\left(320,420\right)$ |
| Convergence temperature for entropy [43] | $T_{S}\sim\text{Unif}\left(320,420\right)$ |

1. Parameter with supporting literature references. [↑](#footnote-ref-2)
2. Shown are the prior distributions which are either Gaussian or uniform distributions. The parameters of the Gaussian distributions are the mean and precision with truncation limits in square brackets. Strain level parameters are subscripted by , domain level parameters by, and membership of strain in domain by . [↑](#footnote-ref-3)
