# Supplementary material for: Universality of Thermodynamic Constants Governing Biological Growth Rates
Source: PLoS One. 2012 Feb 14;7(2):e32003. doi: 10.1371/journal.pone.0032003 (PMC3279425; doi:10.1371/journal.pone.0032003)
Supplement: Table S3 — Domain parameter percent deviation by species. Shown are the maximum and minimum of the strain posterior means for each species, their difference, mid point, and percent deviation. (DOC) [file pone.0032003.s009.doc]

**Table S3. Domain parameter percent deviation by species.**

**Shown are the maximum and minimum of the strain posterior means for each species, their difference, mid point, and percent deviation.**

| **Number of amino acid residues, $n$.** | | | | | | |
| --- | --- | --- | --- | --- | --- | --- |
| **Species** | **no** | **Minimum** | **Maximum** | **Difference** | **Midpoint** | **Deviation** |
| *E. coli* | 13 | 153 | 313 | 160 | 232.9 | 68.8 |
| *L. monocytogenes* | 6 | 149 | 256 | 107 | 202.6 | 53.0 |
| *S. cerevisiae* | 10 | 224 | 340 | 116 | 281.9 | 41.3 |
| *S. gelidimarina* | 3 | 337 | 377 | 40 | 357.2 | 11.3 |
| *S. kudriavzevii* | 4 | 362 | 407 | 45 | 384.5 | 11.7 |
| *S. paradoxus* | 3 | 300 | 302 | 2 | 301.0 | 0.6 |
| *S. thermophilus* | 10 | 171 | 381 | 209 | 276.2 | 75.8 |
| mean | 7 | 242 | 340 | 97 | 290.9 | 37.5 |
| **Enthalpy of activation, .** | | | | | | |
| **Species** | **no** | **Minimum** | **Maximum** | **Difference** | **Midpoint** | **Deviation** |
| *E. coli* | 13 | 62925 | 70436 | 7511 | 66680.9 | 11.3 |
| *L. monocytogenes* | 6 | 57617 | 63582 | 5965 | 60599.3 | 9.8 |
| *S. cerevisiae* | 10 | 25050 | 38668 | 13618 | 31858.8 | 42.7 |
| *S. gelidimarina* | 3 | 50451 | 64437 | 13986 | 57444.0 | 24.3 |
| *S. kudriavzevii* | 4 | 36128 | 44323 | 8195 | 40225.1 | 20.4 |
| *S. paradoxus* | 3 | 26840 | 29604 | 2764 | 28222.2 | 9.8 |
| *S. thermophilus* | 10 | 66002 | 106477 | 40474 | 86239.6 | 46.9 |
| mean | 7 | 46430 | 59647 | 13216 | 53038.5 | 23.6 |
| **Number of non-polar hydrogen atoms per amino acid residue, $N_{\text{ch}}$.** | | | | | | |
| **Species** | **no** | **Minimum** | **Maximum** | **Difference** | **Midpoint** | **Deviation** |
| *E. coli* | 13 | 5.6 | 6.1 | 0.6 | 5.8 | 9.9 |
| *L. monocytogenes* | 6 | 5.6 | 6.1 | 0.5 | 5.9 | 8.8 |
| *S. cerevisiae* | 10 | 5.4 | 5.8 | 0.4 | 5.6 | 6.4 |
| *S. gelidimarina* | 3 | 4.6 | 4.7 | 0.0 | 4.6 | 0.8 |
| *S. kudriavzevii* | 4 | 5.1 | 5.2 | 0.1 | 5.1 | 1.8 |
| *S. paradoxus* | 3 | 5.4 | 5.5 | 0.0 | 5.5 | 0.4 |
| *S. thermophilus* | 10 | 5.4 | 6.1 | 0.7 | 5.8 | 11.6 |
| mean | 7 | 5.3 | 5.6 | 0.3 | 5.5 | 5.7 |

| **Heat capacity changes, ${\Delta C}_{p}$.** | | | | | | |
| --- | --- | --- | --- | --- | --- | --- |
| **Species** | **no** | **Minimum** | **Maximum** | **Difference** | **Midpoint** | **Deviation** |
| *E. coli* | 13 | 64 | 66.6 | 2.7 | 65.3 | 4.1 |
| *L. monocytogenes* | 6 | 63 | 64.0 | 1.4 | 63.3 | 2.2 |
| *S. cerevisiae* | 10 | 64 | 65.0 | 1.5 | 64.2 | 2.3 |
| *S. gelidimarina* | 3 | 48 | 48.7 | 0.6 | 48.4 | 1.2 |
| *S. kudriavzevii* | 4 | 59 | 59.9 | 0.6 | 59.6 | 1.0 |
| *S. paradoxus* | 3 | 63 | 63.2 | 0.3 | 63.1 | 0.5 |
| *S. thermophilus* | 10 | 65 | 66.5 | 1.1 | 65.9 | 1.6 |
| mean | 7 | 61 | 62.0 | 1.2 | 61.4 | 1.9 |
| **Temperature at which denaturation is minimised,** $T_{\text{mes}}$. | | | | | | |
|  | no | Minimum | Maximum | Difference | Mid-range | Deviation |
| *E. coli* | 13 | 297.8 | 301.0 | 3.1 | 299.4 | 1.0 |
| *L. monocytogenes* | 6 | 296.2 | 298.0 | 1.8 | 297.1 | 0.6 |
| *S. cerevisiae* | 10 | 297.3 | 299.1 | 1.8 | 298.2 | 0.6 |
| *S. gelidimarina* | 3 | 272.1 | 273.4 | 1.3 | 272.8 | 0.5 |
| *S. kudriavzevii* | 4 | 291.9 | 292.7 | 0.8 | 292.3 | 0.3 |
| *S. paradoxus* | 3 | 296.6 | 297.0 | 0.4 | 296.8 | 0.1 |
| *S. thermophilus* | 10 | 299.6 | 300.8 | 1.2 | 300.2 | 0.4 |
| mean | 7 | 293.1 | 294.6 | 1.5 | 293.8 | 0.5 |
